# Identification of Alzheimer’s Disease Molecular Subtypes Based on Parallel Large-Scale Sequencing

**DOI:** 10.3389/fnagi.2022.770136

**Published:** 2022-04-28

**Authors:** Meigang Ma, Yuhan Liao, Xiaohua Huang, Chun Zou, Liechun Chen, Lucong Liang, Youshi Meng, Yuan Wu, Donghua Zou

**Affiliations:** ^1^Department of Neurology, The First Affiliated Hospital of Guangxi Medical University, Nanning, China; ^2^Department of Neurology, The Affiliated Hospital of Youjiang Medical University for Nationalities, Baise, China; ^3^Department of Neurology, The Second Affiliated Hospital of Guangxi Medical University, Nanning, China; ^4^Department of Neurology, The Fifth Affiliated Hospital of Guangxi Medical University, Nanning, China

**Keywords:** Alzheimer’s disease, molecular subtype, classifier, diagnosis, parallel sequencing

## Abstract

The incidence of Alzheimer’s disease (AD) is constantly increasing as the older population grows, and no effective treatment is currently available. In this study, we focused on the identification of AD molecular subtypes to facilitate the development of effective drugs. AD sequencing data collected from the Gene Expression Omnibus (GEO) database were subjected to cluster sample analysis. Each sample module was then identified as a specific AD molecular subtype, and the biological processes and pathways were verified. The main long non-coding RNAs and transcription factors regulating each “typing pathway” and their potential mechanisms were determined using the RNAInter and TRRUST databases. Based on the marker genes of each “typing module,” a classifier was developed for molecular typing of AD. According to the pathways involved, five sample clustering modules were identified (mitogen-activated protein kinase, synaptic, autophagy, forkhead box class O, and cell senescence), which may be regulated through multiple pathways. The classifier showed good classification performance, which may be useful for developing novel AD drugs and predicting their indications.

## Introduction

Alzheimer’s disease (AD) is a heterogeneous neurodegenerative disease, which varies in terms of age at dementia onset, genetic risk factors, clinical manifestations, and rates and types of cognitive decline (Lam et al., 2013). The first symptoms of AD are mild memory difficulties, which gradually develop into cognitive dysfunction, personality changes, and language impairment (Zhu et al., 2017). Gradual memory loss and subsequent dementia, as well as neuropathological deposition of senile plaques and neurofibrillary tangles, have been identified as the main clinical symptoms (Giri et al., 2016), while production of amyloid-β (Aβ) and hyperphosphorylation of tau have been observed at the molecular level (Kumar et al., 2015). In addition to Aβ toxicity (O’Brien and Wong, 2011), several processes have been related to the pathogenesis of AD, including hyperphosphorylation of tau protein (Martin et al., 2011), gene mutation, central cholinergic neuron damage, microglia cell activation (Paolicelli et al., 2017), free radical damage, and oxidative stress (Chen and Zhong, 2014). However, the exact disease mechanisms have not yet been elucidated, and they are likely to be multiple and complex.

Recent studies have shown that autophagy defects are often associated with the early stages of AD. Autophagy plays an important role in the production and metabolism of Aβ, while autophagy dysfunction has been found to promote disease progression (Li et al., 2017). Abnormalities in the Wingless-Int (Wnt)/β-catenin pathway have also been related to AD, as the Wnt signaling pathway is involved in the development of neural circuits and the regulation of synaptic transmission and plasticity in adult brain. Its activation is also involved in other pathological disorders, including cancer and neurodegenerative diseases, which reflects its relevance to basic biological processes (Inestrosa and Varela-Nallar, 2014). In AD, the typical Wnt/β-catenin pathway is downregulated, while peroxisome proliferation-activated receptor γ is upregulated (Jia et al., 2019). In particular, Aβ activates glycogen synthase kinase-3β and inhibits the phosphatidylinositol 3-kinase/Akt (PI3K-Akt) signaling pathway, leading to oxidative stress and downregulation of Wnt/β-catenin (Vallee et al., 2017). Additional studies have shown that the integrated coordination of neuronal responses through the PI3-K/Akt pathway affects various functions in AD. For example, activation of Akt may play a therapeutic role in neurodegenerative disease (D’Antoni et al., 2017; Matsuda et al., 2018), while the abnormal, sustained activation of neuronal PI3-K/Akt/mTOR signaling is an early feature of AD (Griffin et al., 2005; O’ Neill, 2013). Long non-coding RNAs (lncRNAs) and transcription factors (TFs) can also regulate AD pathogenesis (Zou C. et al., 2019), which involves downregulation of genes involved in cholinergic synapse transmission (Zhou et al., 2021). Moreover, CXCR 4, EGFR, MAP4K4, and IGF1R may be potential biomarkers and therapeutic targets in AD (Jian et al., 2021). Overall, these studies indicate that multiple mechanisms contribute to AD, leading to distinct molecular subtypes.

Despite advances in AD treatment over the past 20 years, existing drugs mainly target the symptoms, and no method has been found to successfully reduce the accumulation of Aβ in the brain of AD patients (Gao et al., 2016). In addition, early diagnosis and treatment cannot be easily achieved due to the long incubation period of the disease. Given the expanding aging population (Alzheimer’s, 2016), AD prevalence and mortality are constantly increasing, highlighting the need to clarify the mechanisms of AD and develop more effective treatments that can mitigate its effects on patients, their families, and society (Takeda, 2019).

Sequencing technology is widely used as an alternative approach to study complex diseases such as AD. However, research on AD subtypes using bioinformatics analysis has rarely been reported, and most existing reports refer to clinical subtypes of AD, including rapidly progressive AD, posterior cortical atrophy variant, and typical AD of prolonged duration (Qiang et al., 2017). Here, we aimed to identify potential molecular subtypes of AD through bioinformatics analysis using the Gene Expression Omnibus (GEO) database, which includes a large amount of AD-related sequencing data. The results of the study may help guide the development of new AD treatments and provide a basis for further research on the molecular mechanism of the disease.

## Materials and Methods

### Data Collection

Gene expression data related to AD were collected from five different GEO platforms^1^ (Barrett et al., 2013): GPL13669, GPL4372, GPL570, GPL96, and GPL97. Platform GPL13669 included four datasets (GSE29654, GSE29676, GSE39087, and GSE62283), which consisted of 486 AD samples. Platform GPL4372 included the GSE33000 and GSE44772 datasets, comprising 403 AD samples. Platform GPL570 consisted of 13 datasets (GSE4757, GSE5281, GSE9770, GSE16759, GSE18309, GSE28146, GSE28379, GSE29652, GSE48350, GSE53890, GSE66333, GSE84422, and GSE110298), including 314 AD samples. Platform GPL96 included four datasets (GSE1297, GSE4097, GSE12685, and GSE84422), comprising 200 AD samples. Platform GPL97 included only the GSE84422 dataset with 194 AD samples. The GSE85426 dataset of the GPL14550 platform included peripheral blood samples from 90 AD patients and was used to verify the AD subtype classifier.

**FIGURE 1:**
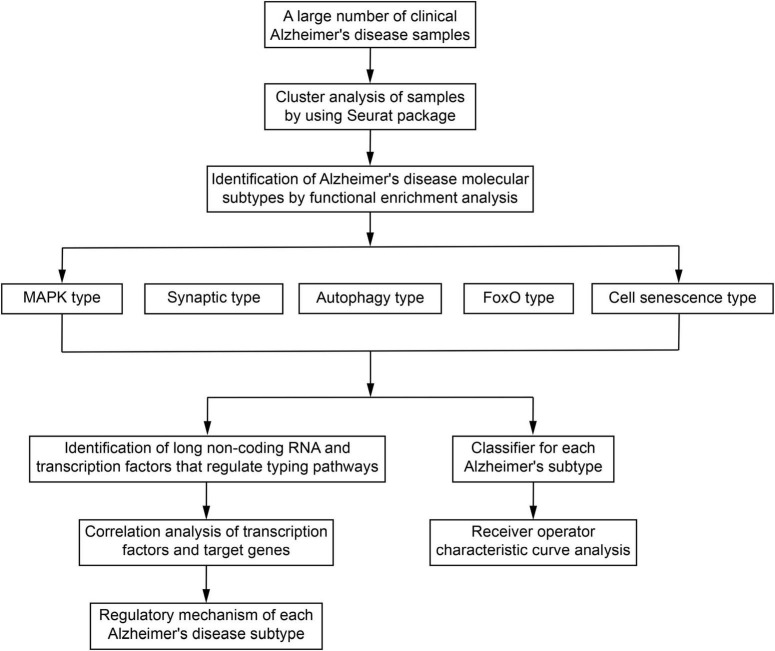
Study flowchart. MAPK, mitogen-activated protein kinase; FoxO, forkhead box class O.

After removing duplicate samples, 1,353 unique AD samples were collected (Supplementary Table 1). The ComBat function of the sva package (Leek et al., 2012) was used to merge the AD samples data from the same platform and remove batch effects. The top 2,000 highly variable genes of samples were then subjected to principal component analysis (PCA) (Supplementary Table 2), and the top 50 principal components (PCs) were extracted in order to perform clustering (Section 2.2). The steps in the identification of molecular subtypes of AD are provided in a flowchart in Figure 1.

### Identification of Potential Alzheimer’s Disease Molecular Subtypes

In order to identify the potential molecular subtypes of AD, the collected AD samples were investigated by cluster analysis using the Seurat package (Butler et al., 2018). The data were first merged with the “IntegrateData” function of the Seurat package and then normalized using the LogNormalize method. For genes detected using multiple probes, the mean value obtained across all probes was considered as the expression value of the corresponding gene. The “FindClusters” function of Seurat package was then applied to identify the different subtype clusters, and the Uniform Manifold Approximation and Projection (UMAP) method was used to visualize the results. The sample modules were also compared using the “FindAllMarkers” function to determine potential marker genes of each cluster and retain the positive genes. The same function was further used to identify genes that were differentially expressed between two specific sample modules. Receiver operating characteristic (ROC) curve analysis was also applied, and the area under the ROC curve (AUC) was calculated for each gene in order to evaluate its accuracy in identifying the target sample module.

### Function and Pathway Enrichment Analysis

The function and pathway enrichment analysis of marker genes (defined as those showing AUC > 0.7) in different subtype clusters was performed using the “enrichGO” and “enrichKEGG” functions of the clusterProfiler package (Yu G. et al., 2012). The subtype clusters were then identified as specific AD molecular subtypes according to the biological processes and pathways in which they were mainly involved.

### Gene Set Variation and Enrichment Analysis

The datasets c5.bp.v6.2.symbols.gmt and c2.cp.kegg.v6.2.symbols.gmt in the MsigDB V6.2 database (Liberzon et al., 2015) were used as reference gene sets, and gene set enrichment analysis (GSEA) was performed using GSEA2-2.2.4 (Java version) (Subramanian et al., 2005). Differences with a nominal *P* < 0.05 were considered statistically significant. Using the GSVA package (Hanzelmann et al., 2013), we calculated gene set variation analysis (GSVA) scores for the biological processes and pathways that involved the marker genes of each sample, and the scores were then visualized as a heat map.

### Construction of a Regulatory Network of Alzheimer’s Disease Molecular Types

Based on the RNAInter database (Lin et al., 2020), we used the hypergeometric test to identify the lncRNAs that interacted with at least two AD sample cluster genes and constructed a comprehensive network of lncRNAs regulating the AD sample modules, which was visualized using Cytoscape software (Shannon et al., 2003).

### Identification of the Regulatory Mechanism of Each Alzheimer’s Disease Molecular Subtype

In order to explore the potential regulatory mechanism of each AD molecular subtype, the lncRNAs and TFs interacting with their pathway genes were extracted using, respectively, the RNAInter and TRRUST (Han et al., 2018) databases. The “cor.test” function was used to calculate the Pearson correlation coefficients between the pathway genes and the TFs. Only TFs with *P* < 0.01 were retained. Based on the identified lncRNAs, TFs, and the main Kyoto Encyclopedia of Genes and Genomes (KEGG) signaling pathways (Kanehisa and Goto, 2000) involved in different AD molecular subtypes, we described the main regulatory mechanisms of the different molecular typing modules.

### Development of the Alzheimer’s Disease Classifier

A total of 1,353 AD samples were randomly divided into a training and a test set in a ratio of 7:3 to ensure an adequate number of samples in the test set. The expression profiles of the marker genes were then extracted to develop an AD classifier model using the “vglm” function in the VGAM package (Yee, 2017). The “predict” function in the VGAM package was used to predict the classification in the training and test sets. Furthermore, an independent dataset (GSE85426) was used to further validate this classifier.

## Results

### Potential Molecular Subtypes of Alzheimer’s Disease

In order to identify potential molecular subtypes of AD, we performed large-scale parallel sequencing and cluster analysis. Before sample typing, all samples were subjected to PCA to remove noise and the top 50 PCs were extracted, leading to massive parallel sequencing data for subtype identification. Unlike the results obtained before removing batch effects (Supplementary Figure 1A), the sample types after removing batch effects were independent of the tissue types of the sequenced samples, while blood and brain tissues (multiple parts) were well distributed (Supplementary Figure 1B). The UMAP maps GPL and GSE also indicated that the typing system was capable of classifying different platforms, sources, and organs. In addition, the expression heat maps of marker genes in sample modules in different clusters showed that the expression patterns of different sample modules were quite heterogeneous (Supplementary Figure 1C).

**FIGURE 2:**
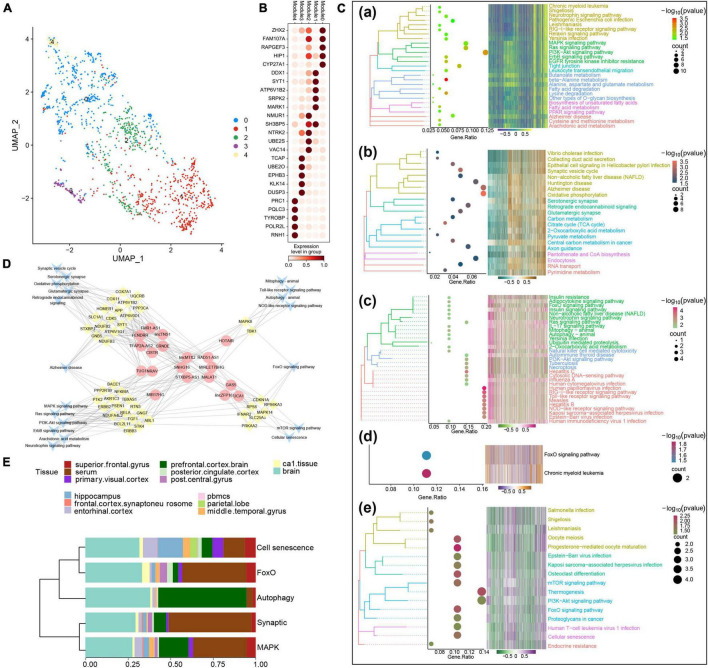
Identification of potential molecular subtypes of Alzheimer’s disease (AD). **(A)** Clinical cohort AD map. Modules 0–4 contain 495, 475, 246, 90, and 47 AD samples, respectively. **(B)** Heat map of marker gene expression in each AD sample module. **(C)** Pathways involving marker genes of different AD sample modules. **(D)** Comprehensive network of long non-coding RNAs (lncRNAs) regulating different AD sample modules, prepared based on a hypergeometric test. **(E)** Diagram of components of each AD clinical sample subtype.

Our analysis identified five AD sample modules, each of which could be related to a potential AD molecular type. Sample modules 0–4 contained 495, 475, 246, 90, and 47 AD samples, respectively (Figure 2A), and their five most significant marker genes were also identified (Figure 2B). Additional enrichment analysis showed that the marker genes of each module were significantly involved in several biological processes such as the regulation of protein kinase B signaling, the regulation of neuron projection development, and protein acylation (Supplementary Figure 2). Moreover, enrichment analysis revealed that the marker genes of each module were significantly involved in various signaling pathways (Figure 2C). Among them, the marker genes of module 0 were involved in neurotrophin signaling, mitogen-activated protein kinase (MAPK) signaling, and AD; marker genes of module 1 were involved in the synaptic vesicle cycle, AD, and serotonergic synapse; marker genes of module 2 were involved in neurotrophin signaling, autophagy, and PI3K-Akt signaling; marker genes of module 3 were involved in forkhead box class O (FoxO) signaling and chronic myeloid leukemia; and the marker genes of module 4 were significantly involved in mammalian target of rapamycin (mTOR) signaling, cellular senescence, and endocrine resistance.

Based on the hypergeometric test, a comprehensive network of lncRNAs that regulated different AD typing pathways was prepared (Figure 2D), and some of the biological processes and pathways enriched in the AD sample modules were verified by GSEA (Supplementary Figure 3). According to the results of the above analyses and the biological processes and pathways in which the marker genes were involved, the five AD sample modules were defined as the following molecular types: the MAPK type (495 samples), involving MAPK/PI3K-Akt/neurotrophin signaling; the synaptic type (475 samples), involving oxidative phosphorylation and synapses; the autophagy type (246 samples), involving Toll, nucleotide oligomerization domain (NOD), FoxO, PI3K-Akt, and autophagy; the FoxO type (90 samples); and the cell senescence type (47 samples), involving mTOR, FoxO, and cell senescence. The various clinical samples showed similar proportions of the various molecular subtypes (Figure 2E). In addition, after Kruskal–Wallis test, the *p*-value of the overall data difference is 0.4781, indicating that there is no obvious correlation between typing and experimental cohort, and they are independent of each other (Supplementary Table 3).

### Mitogen-Activated Protein Kinase-Type Alzheimer’s Disease

**FIGURE 3:**
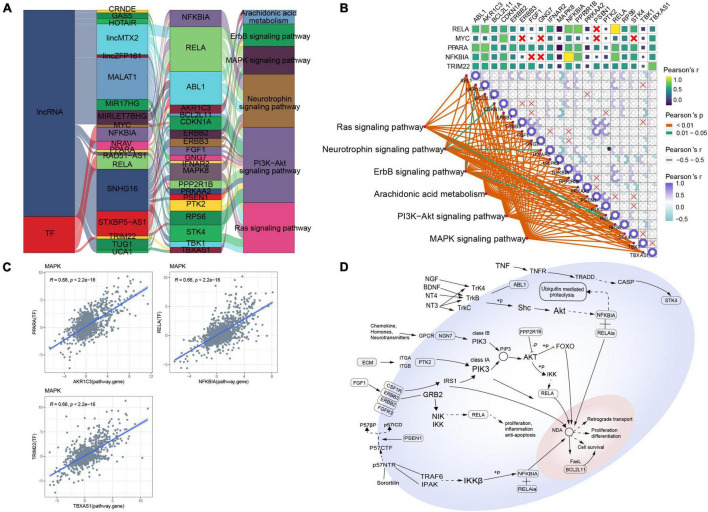
Potential regulatory mechanism of mitogen-activated protein kinase (MAPK)-type Alzheimer’s disease (AD). **(A)** Sankey diagram showing the pathways of MAPK-type AD regulated by long non-coding RNAs (lncRNAs) and transcription factors (TFs) identified using the RNAInter and TRRUST databases. **(B)** Correlation pathway diagram of the gene–pathway, gene–gene, and TF–gene correlations. **(C)** Correlation scatter plot of the correlations between the TFs PPARA, RELA, and TRIM22 and their respective target genes AKR1A3, NFKBIA, and TBXAS1. **(D)** Potential regulatory mechanism of MAPK-type AD.

Several lncRNAs (CRNDE, GAS5, HOTAIR, lincMTX2, lincZFP161, MALAT1, MIR17HG, MIRLET7BHG, NRAV, RAD51-AS1, SNHG16, STXBP5-AS1, TUG1, and UCA1) and TFs (MYC, NFKBIA, PPARA, RELA, and TRIM22) were predicted to regulate MAPK-type AD based on the RNAInter and TRRUST databases, and their regulatory relationship with the corresponding signaling pathways was determined using a Sankey diagram (Figure 3A). As further indicated by the correlation pathway diagram (Figure 3B), most pathways and pathway genes were significantly correlated. A significant positive correlation was also observed between TFs and their target genes (Figure 3C), suggesting that the TFs interacted with multiple pathway genes, affecting the key pathways involved in AD typing. Thus, we propose a potential mechanism of MAPK-type AD in which the TFs PPARA, RELA, and TRIM22 regulate their respective target genes AKR1A3, NFKBIA, and TBXAS1, ultimately regulating MAPK and other signaling pathways (Figure 3D).

### Synaptic-Type Alzheimer’s Disease

**FIGURE 4:**
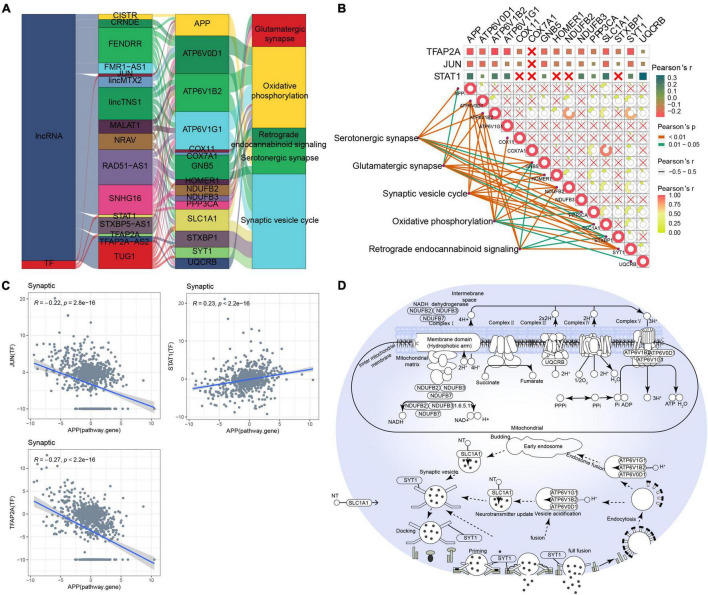
Potential regulatory mechanism of synaptic-type Alzheimer’s disease (AD). **(A)** Sankey diagram showing the pathways of synaptic-type AD regulated by long non-coding RNAs (lncRNAs) and transcription factors (TFs) identified by the RNAInter and TRRUST databases. **(B)** Correlation pathway diagram of the gene–pathway, gene–gene, and TF–gene correlations. **(C)** Correlation scatter plot of the correlations between the TFs JUN, STAT1, and TFAP2A and the target gene APP. **(D)** Potential regulatory mechanism of synaptic-type AD.

As for MAPK-type AD, relationships between lncRNAs/TFs and pathways regulating synaptic-type AD were determined using data from the RNAInter and TRRUST databases (Figure 4A). Several lncRNAs (CISTR, CRNDE, FENDRR, FMR1-AS1, lincMTX2, lincTNS1, MALAT1, NRAV, RAD51-AS1, SNHG16, STXBP5-AS1, TFAP2A-AS2, and TUG1) and TFs (JUN, STAT1, and TFAP2A) were predicted to interact with pathway genes, regulating the main pathway of synaptic-type AD. However, the correlation between most pathway genes was not significant (Figure 4B), and the correlation between JUN and its target pathway gene APP was weak (Figure 4C), implying that the main pathway of synaptic-type AD may involve several regulatory mechanisms. Based on these findings, we propose a potential mechanism for synaptic-type AD involving primarily pathways related to synaptic function, such as oxidative phosphorylation and the synaptic vesicle cycle (Figure 4D).

### Forkhead Box Class O-Type Alzheimer’s Disease

**FIGURE 5:**
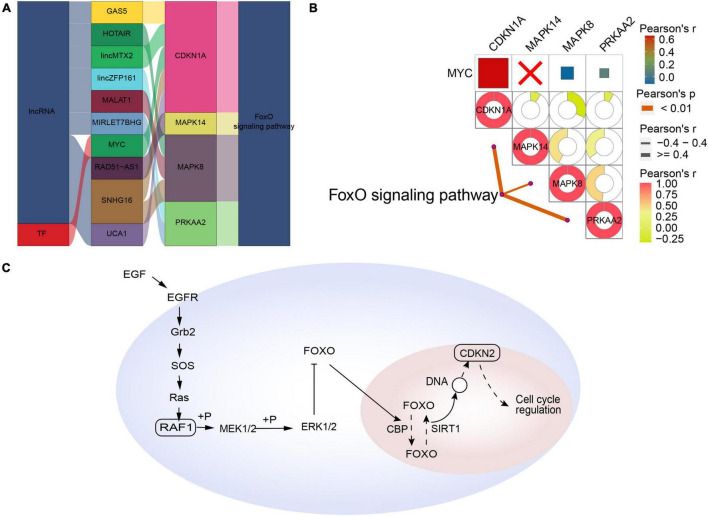
Potential regulatory mechanism of forkhead box class O (FoxO)-type Alzheimer’s disease (AD). **(A)** Sankey diagram showing the pathway of FoxO-type AD regulated by long non-coding RNAs (lncRNAs) and transcription factors (TFs) identified by the RNAInter and TRRUST databases. **(B)** Correlation pathway diagram of the gene–pathway, gene–gene, and TF–gene correlations. **(C)** Potential regulatory mechanism of FoxO-type AD.

Forkhead box class O-type AD was regulated by the interaction of lncRNAs (GAS5, HOTAIR, lincMTX2, lincZFP161, MALAT1, MIRLET7BHG, RAD51-AS1, SNHG16, and UCA1) and the TF MYC with the corresponding pathway genes (Figure 5A). In contrast to synaptic-type AD, the pathway genes CDKN1A, MAPK14, MAPK8, and PRKAA2 correlated significantly with the corresponding FoxO signaling pathway (Figure 5B). Therefore, we propose a mechanism of FoxO-type AD involving regulation by MYC (Figure 5C).

### Cellular Senescence-Type Alzheimer’s Disease

**FIGURE 6:**
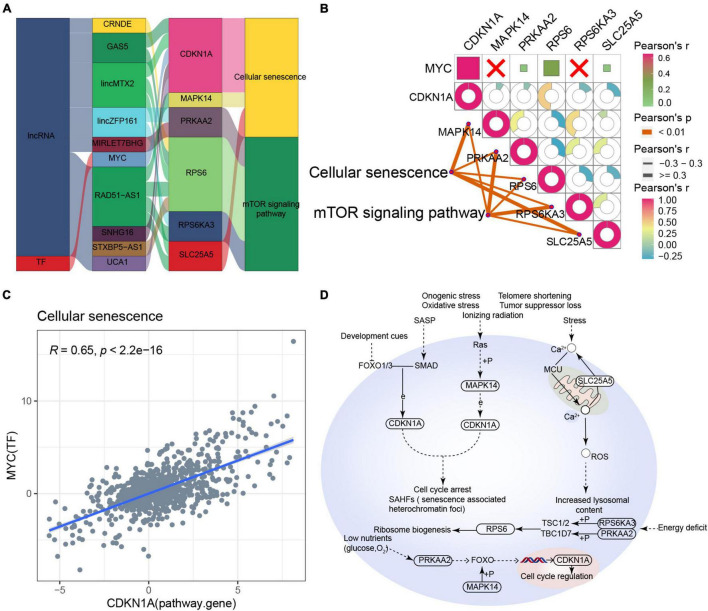
Potential regulatory mechanism of cellular senescence-type Alzheimer’s disease (AD). **(A)** Sankey diagram showing the pathway of cellular senescence-type AD regulated by long non-coding RNAs (lncRNAs) and transcription factors (TFs) identified by the RNAInter and TRRUST databases. **(B)** Correlation pathway diagram of the gene–pathway, gene–gene, and TF–gene correlations. **(C)** Correlation scatter plot of the correlation between the TF MYC and the target gene CDKN1A. **(D)** Potential regulatory mechanism of cellular senescence-type AD.

Based on the RNAInter and TRRUST databases, we found that the main pathway of cellular senescence-type AD was regulated by the interaction of lncRNAs (CRNDE, GAS5, lincMTX2, lincZFP161, MIRLET7BHG, RAD51-AS1, SNHG16, STXBP5-AS1, and UCA1) and MYC with various pathway genes (Figure 6A). Moreover, the cellular senescence and mTOR signaling pathways correlated significantly with the corresponding pathway genes, while MYC correlated significantly with its target gene CDKN1A (Figures 6B,C), leading us to propose an underlying regulatory mechanism for cellular senescence-type AD (Figure 6D).

### Autophagic-Type Alzheimer’s Disease

**FIGURE 7:**
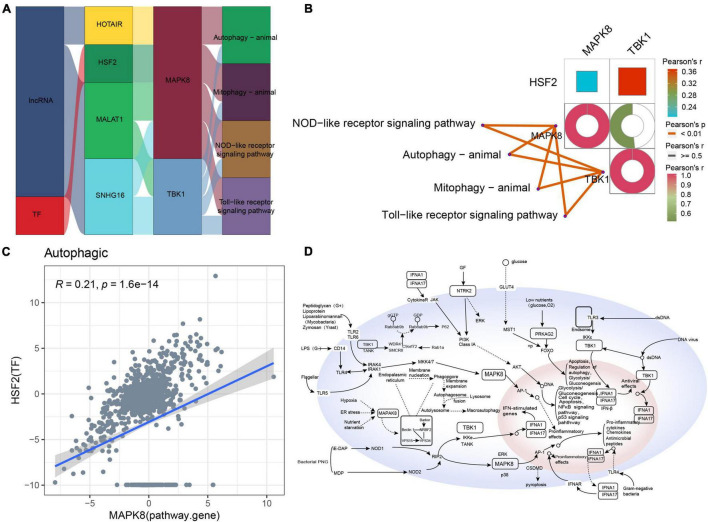
Potential regulatory mechanism of autophagic-type Alzheimer’s disease (AD). **(A)** Sankey diagram showing the pathway of autophagic-type AD regulated by long non-coding RNAs (lncRNAs) and transcription factors (TFs) identified by the RNAInter and TRRUST databases. **(B)** Correlation pathway diagram of the gene–pathway, gene–gene, and TF–gene correlations. **(C)** Correlation scatter plot of the correlation between the TF HSF2 and the target gene MAPK8. **(D)** Potential regulatory mechanism of autophagic-type AD.

The Sankey diagram obtained for autophagic-type AD indicated that the lncRNAs HOTAIR, MALAT1, and SNHG16 and the TF HSF2 may target pathway genes such as MAPK8 and TBK1 to affect key pathways such as autophagy, mitophagy, NOD-like receptor signaling, and Toll-like receptor signaling (Figure 7A). Moreover, these pathway genes correlated significantly with the corresponding pathways (Figure 7B). However, the correlation between MAPK8 and HSF2 was poor (Figure 7C). Based on these results, we propose a potential regulatory mechanism of autophagic-type AD (Figure 7D).

### AD Classification Based on the Five Identified Molecular Subtypes

**FIGURE 8:**
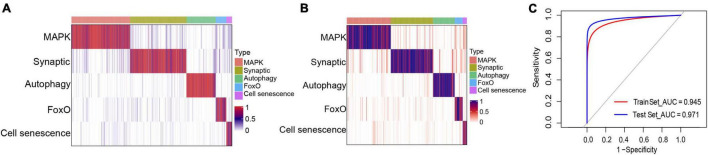
Classifier construction for the identification of different molecular subtypes of Alzheimer’s disease. **(A,B)** Matrix heat maps showing the classification performance with the **(A)** training and **(B)** test datasets. **(C)** Receiver operating characteristic curve analysis of the training and test datasets.

Based on the marker genes corresponding to each molecular typing module, we prepared a classifier for AD molecular typing, which showed good classification performance in both the training and test datasets (Figures 8A,B). ROC curve analysis was further applied to evaluate the classifier’s diagnostic performance, which indicated that both the training and test datasets had good prediction performance (Figure 8C). Additionally, the independent dataset showed that both the training and test datasets could generate these five modules (Supplementary Figure 4).

## Discussion

Although significant progress has been made in clarifying the pathogenesis and symptoms of AD (Gao et al., 2016), no effective treatment is currently available except for drugs that only alleviate symptoms. Therefore, the identification of potential AD molecular typing modules may contribute not only to the prediction of their molecular targets but also to the development of new drugs for AD. In this study, we identified five sample clustering modules using sequencing data from 1,353 AD samples, and the markers of each module were extracted for further functional and enrichment analyses. According to their involvement in different biological processes and pathways and based on literature findings, the five sample clustering modules were associated with five potential molecular subtypes: MAPK, synaptic, autophagy, FoxO, and cell senescence.

MAPKs include c-Jun NH2 terminal kinase, p38 MAPK, and extracellular signal-regulated kinase, which regulate various cell activities, including proliferation, differentiation, apoptosis, inflammation, and innate immunity (Kim and Choi, 2015). It has been reported that neuroinflammation is a common pathway leading to many potential AD pathologies, while p38 MAPK is known as an important target of chronic inflammation. *In vivo* and *in vitro* studies on AD agents (Lee and Kim, 2017; Kheiri et al., 2018) suggest that the inhibition of p38 MAPK may be a potential treatment for MAPK-type AD. Moreover, a number of lncRNAs and TFs that may regulate MAPK-type AD have been identified in our study, some of which have well-defined roles in AD, such as GAS5, which may promote the recovery of neurons and cholinergic nervous system (Zhao et al., 2019), MALAT1 has a neuroprotective effect (Li et al., 2020), and PPARA regulates autophagy to clear Aβ (Luo et al., 2020). We also found bioinformatic evidence that these lncRNAs and TFs ultimately regulate MAPK and other signaling pathways by regulating their respective target genes. For example, lincMTX2, MIRLET7BHG, and STXBP5-AS1 can regulate MAPK signaling pathway by regulating RELA, so as to promote cell differentiation, inflammation, and anti-apoptosis. Thus, the MAPK signaling pathway may be a therapeutic target in AD.

The microtubule-associated tau protein and Aβ may interact at a post-synaptic stage to play a key role in AD; tau-dependent synaptic dysfunction is a major mechanism of AD pathogenesis (Ittner and Ittner, 2018). Synapse loss has also been identified as the strongest pathological factor related to cognitive decline in AD cases, suggesting that synaptic degeneration is critical in the pathogenesis of dementia and that synaptic failure may be a manifestation of synaptic-type AD (Chen et al., 2019). Furthermore, we found that the pathways involved in synaptic-type AD may be regulated by multiple lncRNAs and TFs. At serotonergic synapses, CISTR, FENDRR, JUN, and STAT1 may protect postsynaptic neurons by targeting APP, so low expression of these genes may inhibit neuroprotection. In the synaptic vesicle cycle, TUG1 may regulate the release of synaptic vesicles by targeting SLC1A1, STXBP1, and SYT1, thereby regulating the communication between neurons. In oxidative phosphorylation, TUG1, NRAV, and SNHG16 promote expression of NADH dehydrogenase and cytochrome oxidase by targeting COX111, NDUFB2, and NDUFB3. Targeting SYT1 has been shown to increase synaptic and memory defects (Shi et al., 2020). Furthermore, accumulated tau stimulates Aβ production by upregulating STAT1-BACE1 signaling (Zhang et al., 2021), suggesting that these lncRNAs and TFs may be potential targets in synaptic-type AD.

Oxidative stress is another important determinant in the pathogenesis of AD (Manolopoulos et al., 2010). FoxO is not only involved in the response of cells to oxidative stress, but it also plays a vital role in determining the survival of various cell types in the nervous system by inducing apoptosis and autophagy pathways, while controlling the proliferation and differentiation of stem cells (Maiese, 2016). Therefore, FoxO may also be involved in a potential molecular type of AD. Our study suggests that FoxO-type AD genes such as CDKN1A, MAPK14, MAPK8, and PRKAA2 may be regulated by lncRNAs and TFs such as GAS5, HOTAIR, and MYC. In particular, CDKN1A, which together with interacting zinc finger protein 1 (CIZ1) plays a key role in DNA replication and cell cycle progression through the G1/S checkpoint (Khan et al., 2018), may contribute to neurodegenerative diseases. Therefore cell cycle dysfunction may be a potential target in FoxO-type AD.

Aging is the main risk factor for most neurodegenerative diseases, including AD and Parkinson’s disease (Hou et al., 2019). Cellular senescence has been observed in brain aging and neurodegenerative diseases, suggesting that they may promote dysfunction of the central nervous system (Baker and Petersen, 2018). A recent study has also reported the involvement of Aβ-induced oligodendrocyte progenitor cellular senescence in neuroinflammation and cognitive deficits in AD (Zhang et al., 2019), consistent with the existence of a cell senescence type of AD. By cross-correlating our bioinformatic analyses with clinical information for the AD patients, we confirmed that cellular senescence-type AD was associated with older age (Supplementary Figure 5). Moreover, our study revealed numerous regulators in cellular senescence-type AD, including CRNDE, GAS5, lincMTX2, and lincZFP161, which regulate pathways involving CDKN1A, MAPK14, and PRKAA2. For example, during cellular senescence, CDKN1A can be targeted to indirectly regulate cell cycle arrest and senescence-associated heterochromatin foci. In the mTOR signaling pathway, targeting RPS6 can indirectly regulate multiple functional processes such as cellular protein synthesis, glycolipid metabolism, and autophagy regulation. Therefore these pathways may be therapeutic targets in cellular senescence-type AD.

Autotrophy is an important degradation pathway that eliminates abnormal protein aggregation in mammalian cells and is associated with protein homeostasis and neuronal health (Li et al., 2017). Autophagy defects can lead to neurodegeneration in many diseases, including AD, and recent reports have suggested that they may also be the main manifestation of autophagy-type AD (Zare-Shahabadi et al., 2015; Zou D. et al., 2019). The present study found that HOTAIR, HSF2, MALAT1, and SNHG16 may regulate autophagy and mitophagy by targeting MAPK8 and TBK1. TKB1 has already been linked to inflammatory responses (Yu T. et al., 2012), which is one of the causes of many chronic diseases including AD, and our study identified potential regulators of its expression, which may be therapeutic targets in this AD subtype.

Although we used marker genes from five candidate AD molecular typing modules to accurately type AD in clinical samples, our study still has some limitations, including the fact that the sample was relatively small, which may affect the quality of statistical analysis. In addition, our results were obtained mainly through bioinformatics analysis, so whether molecular AD subtypes are associated with clinical AD progression remains unclear. Therefore, our findings should be verified and extended in future studies with larger samples that draw from additional clinical databases and verify the *in silico* findings in molecular experiments.

## Conclusion

Based on marker genes, we identified five candidate AD molecular typing modules and named them MAPK, synaptic, autophagy, FoxO, and cell senescence types. Accurate AD molecular typing may be useful for predicting their molecular targets and developing novel AD drugs.

## Data Availability Statement

The datasets presented in this study can be found in online repositories. The names of the repository/repositories and accession number(s) can be found in the article/Supplementary Material.

## Author Contributions

DZ and YW conceived and designed the study. MM, YL, and XH performed the experiments. All authors analyzed the data, prepared the figures and tables, wrote and reviewed the manuscript, and approved its submission.

## Conflict of Interest

The authors declare that the research was conducted in the absence of any commercial or financial relationships that could be construed as a potential conflict of interest.

## Publisher’s Note

All claims expressed in this article are solely those of the authors and do not necessarily represent those of their affiliated organizations, or those of the publisher, the editors and the reviewers. Any product that may be evaluated in this article, or claim that may be made by its manufacturer, is not guaranteed or endorsed by the publisher.

## Footnotes

1
http://www.ncbi.nlm.nih.gov/geo

## Supplementary Material

Supplementary Figure 1 Principal component analysis (PCA) of data and expression of marker genes in different sample modules. **(A)** PCA of each dataset and platform before removing batch effects. **(B)** PCA of each dataset and platform after removing batch effects. **(C)** The expression heat maps of the marker genes of the sample modules in different clusters.

Supplementary Figure 2 Involvement of the marker genes of Alzheimer’s disease modules in various biological processes: **(A)** module 0, **(B)** module 1, **(C)** module 2, **(D)** module 3, and **(E)** module 4.

Supplementary Figure 3 Gene set enrichment analysis showing **(A)** biological processes and **(B)** pathways enriched in different Alzheimer’s disease sample modules: **(a)** module 0, **(b)** module 1, **(c)** module 2, **(d)** module 3, and **(e)** module 4.

Supplementary Figure 4 Validation of the Alzheimer’s disease classifier using an independent dataset.

Supplementary Figure 5 Comparison of age of cellular senescence-type AD samples with other types.

Supplementary Table 1 Clinical information of patient samples included in this study.

Supplementary Table 2 Feature loadings of TOP2 principal components.

Supplementary Table 3 The numbers of each subtype in each data set.

## Abbreviations

AD: Alzheimer’s disease
A β: hyperphosphorylation of amyloid β
AUC: area under the ROC curve
FoxO: forkhead box class O
GEO: Gene Expression Omnibus
GSEA: gene set enrichment analysis
GSVA: gene set variation analysis
KEGG: Kyoto Encyclopedia of Genes and Genomes
lncRNAs: long non-coding RNAs
MAPK: mitogen-activated protein kinase
mTOR: mammalian target of rapamycin
NOD: nucleotide oligomerization domain
PCA: principal component analysis
PI3K-Akt: phosphatidylinositol 3-kinase/Akt
ROC: receiver operating characteristic
TFs: transcription factors
UMAP: Uniform Manifold Approximation and Projection
Wnt: Wingless-Int.
