# Supplementary figures and images for: Identification of Alzheimer’s Disease Molecular Subtypes Based on Parallel Large-Scale Sequencing
Source: Front Aging Neurosci. 2022 Apr 28;14:770136. doi: 10.3389/fnagi.2022.770136 (PMC9112923; doi:10.3389/fnagi.2022.770136)

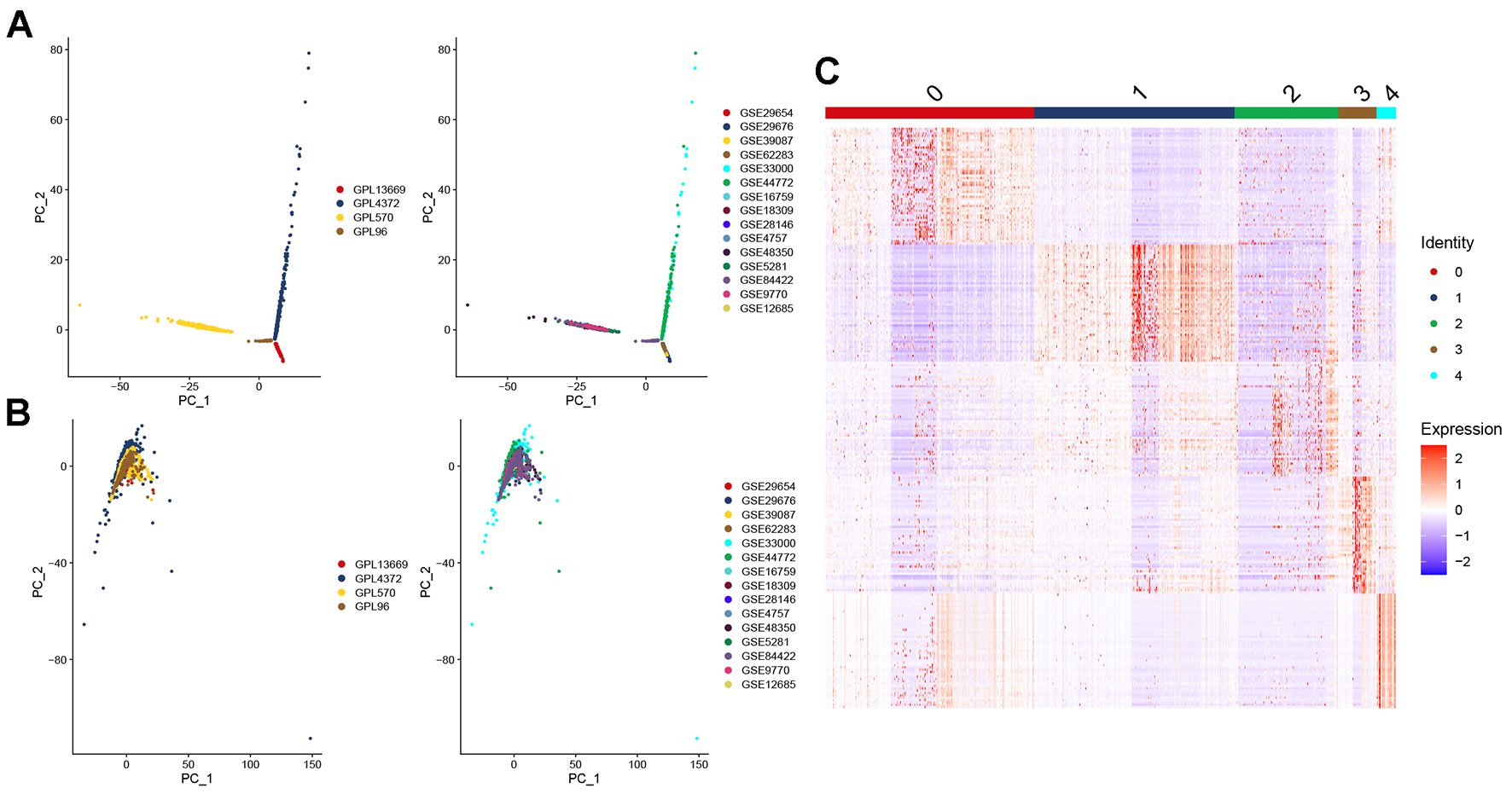

Supplement: Supplementary Figure 1 — Principal component analysis (PCA) of data and expression of marker genes in different sample modules. (A) PCA of each dataset and platform before removing batch effects. (B) PCA of each dataset and platform after removing batch effects. (C) The expression heat maps of the marker genes of the sample modules in different clusters. [file Image_1.TIF]

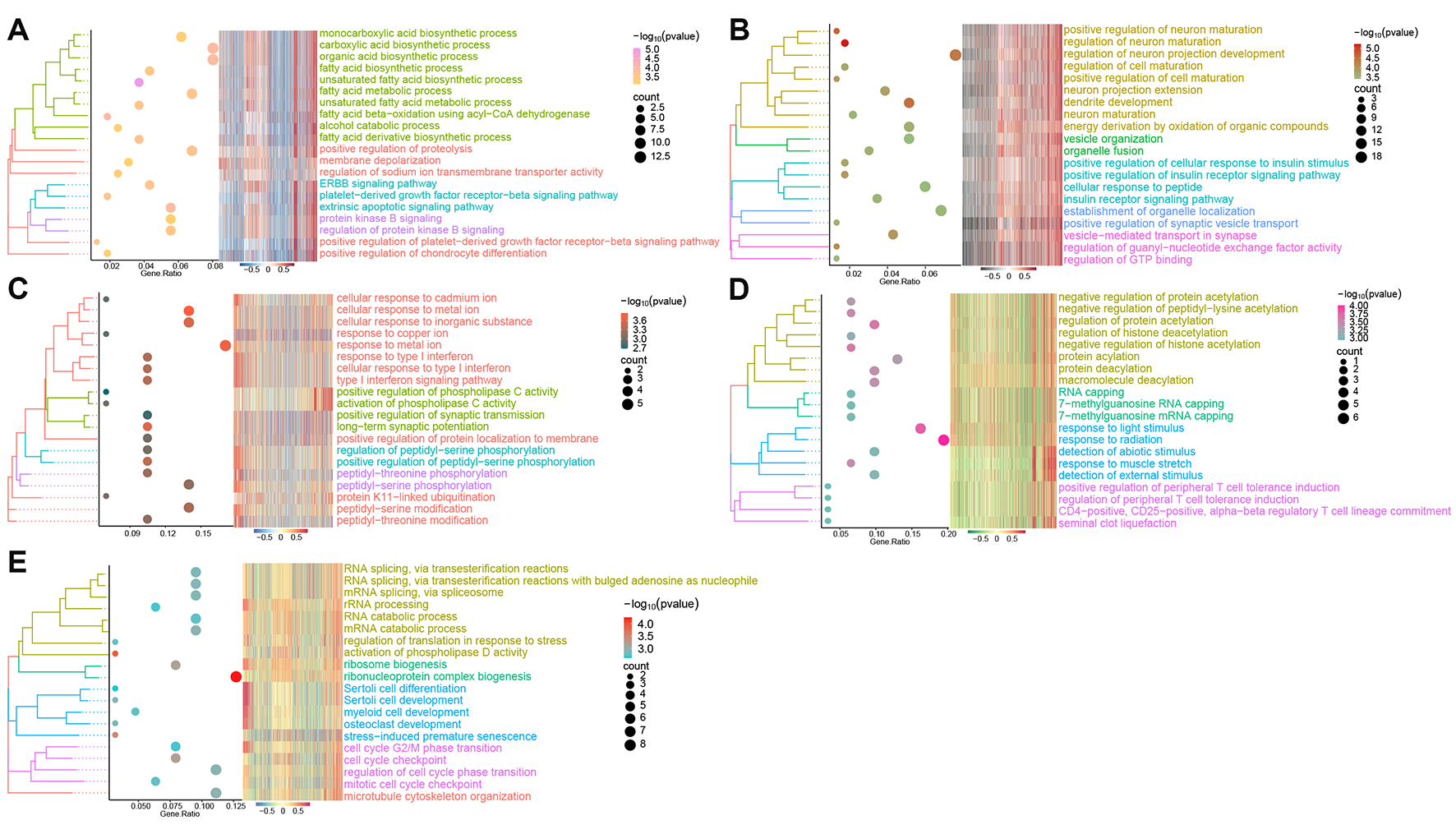

Supplement: Supplementary Figure 2 — Involvement of the marker genes of Alzheimer’s disease modules in various biological processes: (A) module 0, (B) module 1, (C) module 2, (D) module 3, and (E) module 4. [file Image_2.PNG]

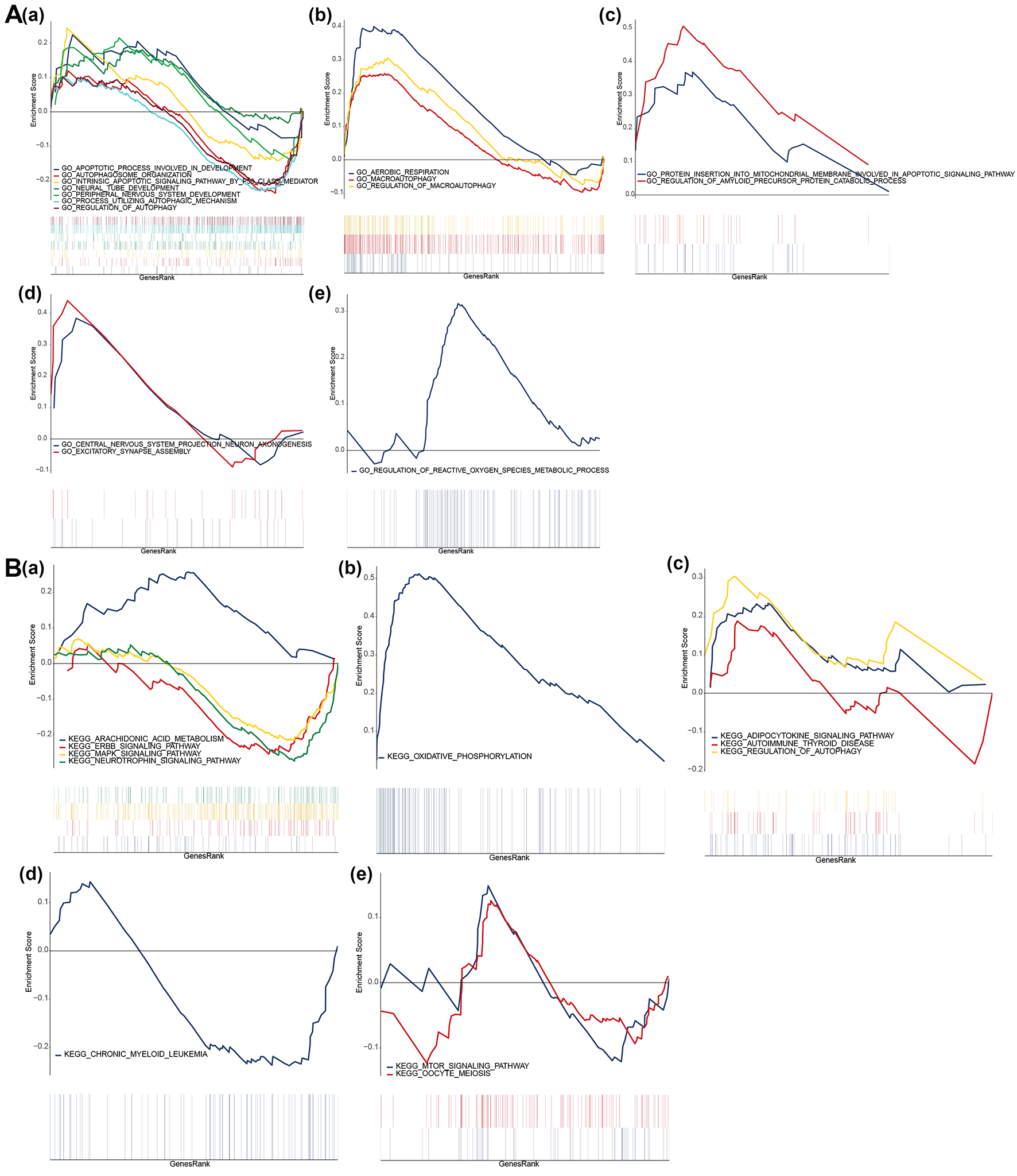

Supplement: Supplementary Figure 3 — Gene set enrichment analysis showing (A) biological processes and (B) pathways enriched in different Alzheimer’s disease sample modules: (a) module 0, (b) module 1, (c) module 2, (d) module 3, and (e) module 4. [file Image_3.PNG]

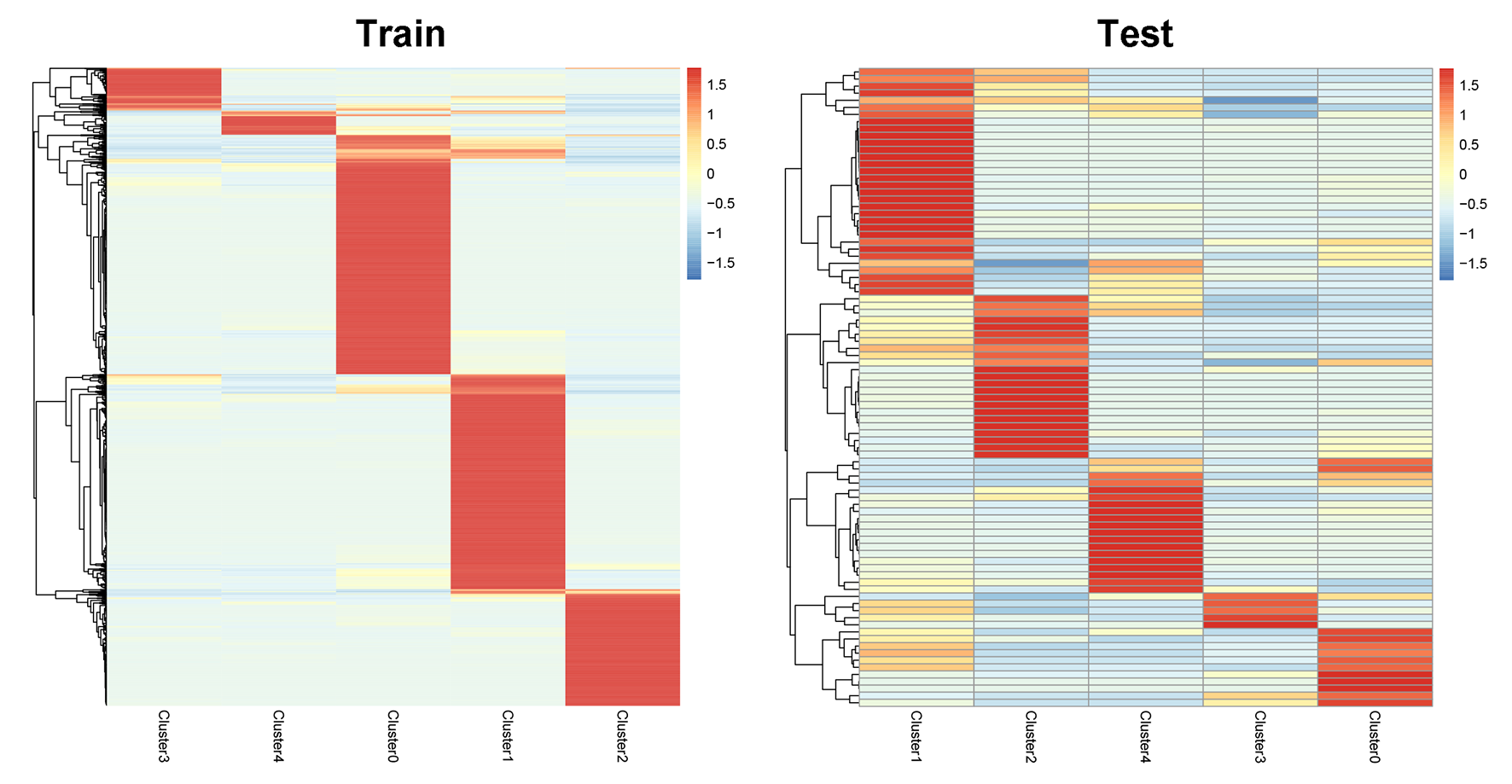

Supplement: Supplementary Figure 4 — Validation of the Alzheimer’s disease classifier using an independent dataset. [file Image_4.PNG]

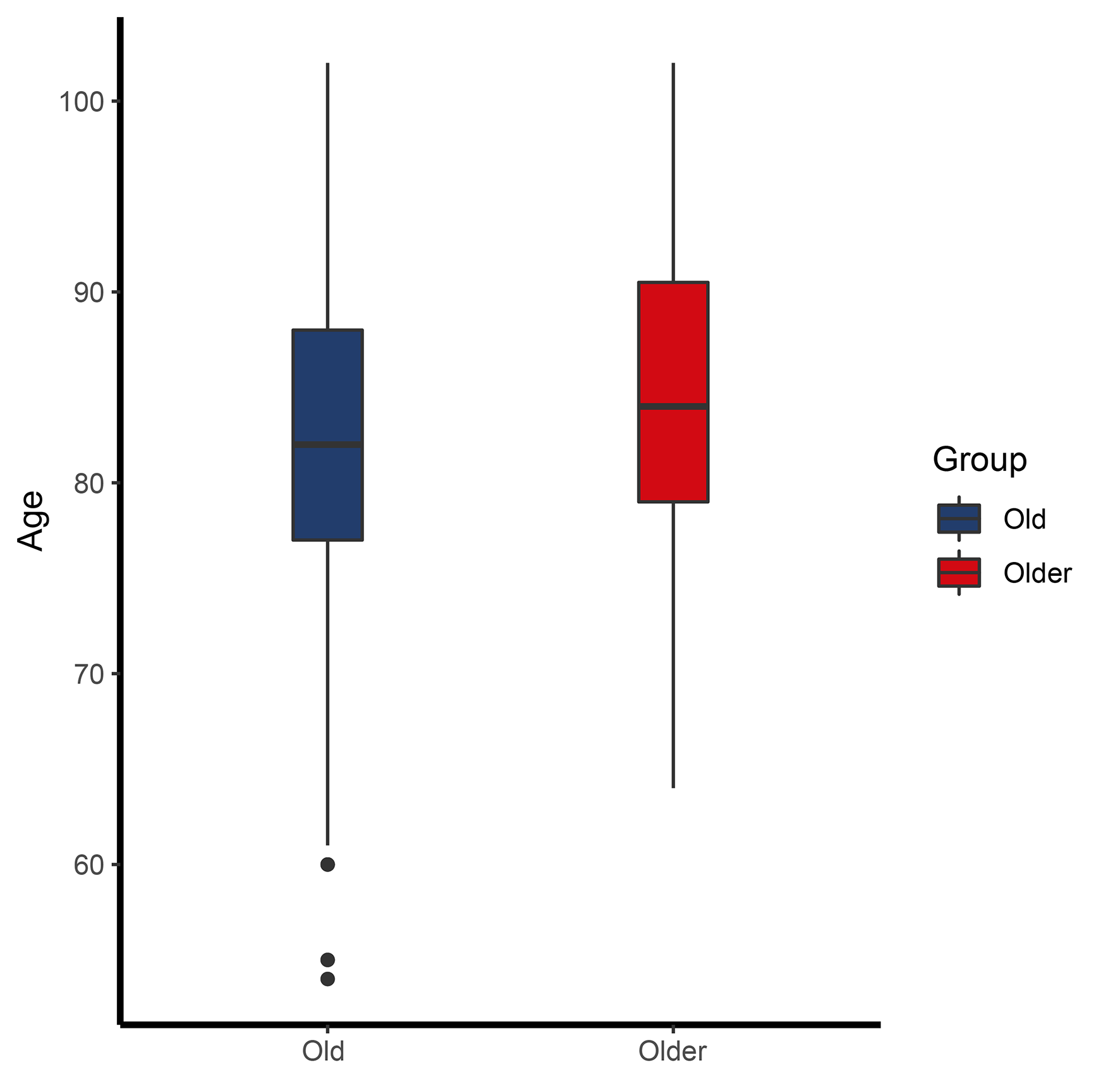

Supplement: Supplementary Figure 5 — Comparison of age of cellular senescence-type AD samples with other types. [file Image_5.PNG]
